# Sapovirus in Adults in Rehabilitation Center, Upper Austria

**DOI:** 10.3201/eid1607.091789

**Published:** 2010-07

**Authors:** Claudia Mikula, Burkhard Springer, Sandra Reichart, Katharina Bierbacher, Alfred Lichtenschopf, Marina Hoehne

**Affiliations:** Author affiliations: Austrian Agency for Health and Food Safety, Graz, Austria (C. Mikula, B. Springer, K. Bierbacher);; Public Health Authority Upper Austria, Linz, Austria (S. Reichart);; Rehabilitation Center Weyer, Weyer, Austria (A. Lichtenschopf);; Robert Koch-Institute, Berlin, Germany (M. Hoehne)

**Keywords:** Sapovirus, Austria, disease outbreaks, adults, epidemiology, gastroenteritis, viruses, sequence analysis, caliciviridae

**To the Editor:** Contrary to norovirus (NoV) infections, *sapovirus (SaV)* is believed to affect mainly young children (*1*), although recent studies show that SaV is present in all age groups (*2**,**3*). SaV has been classified into 5 genogroups, of which GI, GII, GIV, and GV affect humans (*4*). SaV can be transmitted in various ways, including person-to-person fecal–oral route, by aerosol, and by consumption of contaminated food or water (*5*). Outbreaks of SaV have been reported in various settings but are less common than NoV outbreaks (*1**,**6*).

During October 2–7, 2008, an outbreak of gastroenteritis occurred in a rehabilitation center in Upper Austria. Signs including diarrhea, vomiting, and fever developed in 21 adult patients and 12 staff members. The observed signs and the likely incubation period initially suggested NoV as the cause of the outbreak. Stool specimens of 10 patients were collected and submitted to the Institute for Medical Microbiology and Hygiene in Graz.

Along with microbiologic investigations, infection control measures were introduced by local authorities on each affected ward. The earliest reported onset of illness was on October 2, 2008, in a 52-year-old woman on the third floor. The next day 2 additional patients on the same floor and 1 member of the kitchen staff showed symptoms. Another 7 patients, on 3 different floors, and 2 of the medical staff suffered from symptoms the following day (October 4). The outbreak peaked with 11 cases 3 days after the initial episode of vomiting. An additional 9 persons became infected (5 patients, a doctor, janitor, psychotherapist, and kitchen worker) within the following 2 days. The affected patients were predominantly elderly; mean age was 54 years (range 20–81 years, male:female ratio 1:0.83). Clinical signs continued for 24 hours.

Routine microbiologic cultures for enteric bacterial pathogens were performed and showed negative results. A NoV-specific 1-tube real-time PCR assay (LightCycler 2.0; Roche Applied Science, Mannheim, Germany) with primers/probes reported by Hoehne et al*.* (*7*) and ELISAs (Ridascreen; R-Biopharm AG, Darmstadt, Germany) were conducted to detect rotavirus and adenovirus antigen. All tests yielded negative results. Subsequently, 5 of 10 samples were submitted to the Robert Koch Institute in Berlin for further investigation. SaV was identified in 4 samples by using the reverse transcription–PCR described by Oka et al. (*8*); mean viral load was 2.65 × 10^8^ RNA copies/g stool (range 3.3 × 10^7^–7.7 × 10^8^ copies/g stool). Direct sequencing of the appropriate amplification product showed 100% identical nucleotide sequences, which indicated 1 causative strain.

Retrospective testing of the 10 specimens showed 9 SaV-positive samples. Subsequently, genotyping was conducted by using a 1,130-bp amplification product of the polyprotein gene open reading frame 1 spanning the recombination site at the junction between the polymerase gene and the capsid gene. One specimen was amplified by RT-nested PCR with sense primer SV 53a (5′-TAGACTACAGCAAGTGGGA-3′, nt position 4356–4374), antisense primer SV 63 (5′-ACACCATGTTGGACACGCTGC-3′, nt position 6901–6881), and SuperScript III One-Step RT-PCR System with Platinum Taq High Fidelity (Invitrogen, Paisley, UK) for the first-round PCR. For the second-round PCR, the HotStarTaq Master Mix Kit (QIAGEN, Hilden, Germany) and primer SV 55a (5′-CCMTCKGGCATGCCATTCAC-3′, nt position 4529–4548) and SV60 (5′-ATGTTAAATGTGATAGGATCCAC-3′, nt position 5658–5636) were used (nucleotide positions according to GenBank accession no. DQ058829).

Phylogenetic analysis of the second-round PCR product (GenBank accession no. GU724600) showed 97% nucleotide identity to the strain Angelholm/SW278/2004/SE (GenBank accession no. DQ125333), which is a known intergenogroup recombinant virus (II.2/ IV) as seen in Japan and Sweden (*9*). Thus, by sequence analysis of the polymerase region, our strain Graz1561/2008/Austria was grouped into genogroup II; the capsid region belonged to genogroup IV.

Diarrhea and vomiting were the most common signs in patients, 97% and 73%, respectively. Fever was recorded for only 1 case-patient. Our findings are consistent with those of SaV outbreak studies in adults reported by Johansson et al. (*3*), who reported diarrhea in 72% and vomiting in 56% of the case-patients they studied.

The new genetic background of the recombinant virus may have enhanced host susceptibility by evading the immune response and is therefore able to affect adults. Our study shows that SaV causes outbreaks of gastroenteritis in adults; consequently, the role of SaV in the adult population should be reconsidered. We suggest that diagnostics for SaV should be included in the study of gastroenteritis outbreaks in adults, especially when clinical signs suggest NoV as the causative agent but no diagnostic confirmation can be achieved.
